# Bortezomib-based induction, high-dose melphalan and lenalidomide maintenance in myeloma up to 70 years of age

**DOI:** 10.1038/s41375-020-0976-9

**Published:** 2020-07-20

**Authors:** Elias K. Mai, Kaya Miah, Uta Bertsch, Jan Dürig, Christof Scheid, Katja C. Weisel, Christina Kunz, Markus Munder, Hans-Walter Lindemann, Maximilian Merz, Dirk Hose, Anna Jauch, Anja Seckinger, Steffen Luntz, Sandra Sauer, Stephan Fuhrmann, Peter Brossart, Ahmet Elmaagacli, Martin Goerner, Helga Bernhard, Martin Hoffmann, Marc S. Raab, Igor W. Blau, Mathias Hänel, Axel Benner, Hans J. Salwender, Hartmut Goldschmidt, for the German-speaking Myeloma Multicenter Group (GMMG)

**Affiliations:** 1 Department of Internal Medicine V, University Hospital Heidelberg, Heidelberg, Germany; 2 National Center for Tumor Diseases (NCT) Heidelberg, Heidelberg, Germany; 3 Division of Biostatistics, German Cancer Research Center (DKFZ), Heidelberg, Germany; 4 Department of Hematology, University Clinic Essen, Essen, Germany; 5 Department of Internal Medicine I, University Hospital Cologne, Cologne, Germany; 6 Department of Oncology, Hematology and Bone Marrow Transplantation with Section of Pneumology, University Medical Center Hamburg-Eppendorf, Hamburg, Germany; 7 Department of Hematology, Oncology and Immunology, University Hospital Tübingen, Tübingen, Germany; 8 Institute of Child Nutrition, Max Rubner Institute, Federal Research Institute of Nutrition and Food, Karlsruhe, Germany; 9 Department of Internal Medicine III, University Medical Center Mainz, Mainz, Germany; 10 Department of Hematology and Oncology, Katholisches Krankenhaus Hagen, Hagen, Germany; 11 Institute of Human Genetics, University of Heidelberg, Heidelberg, Germany; 12 Coordination Centre for Clinical Trials, University Hospital Heidelberg, Heidelberg, Germany; 13 Department of Hematology and Oncology, Helios Hospital Berlin Buch, Berlin, Germany; 14 University Hospital Bonn, Bonn, Germany; 15 Department of Hematology and Oncology, Asklepios Hospital Hamburg St. Georg, Hamburg, Germany; 16 Department of Hematology, Oncology and Palliative Care, Klinikum Bielefeld, Bielefeld, Germany; 17 Department of Internal Medicine V, Klinikum Darmstadt, Darmstadt, Germany; 18 Medical Clinic A, Klinikum Ludwigshafen, Ludwigshafen, Germany; 19 Medical Clinic, Charité University Medicine Berlin, Berlin, Germany; 20 Department of Internal Medicine III, Klinikum Chemnitz, Chemnitz, Germany; 21 Department of Hematology and Oncology, Asklepios Hospital Hamburg Altona, Hamburg, Germany

**Keywords:** Myeloma, Randomized controlled trials, Stem-cell research

## Abstract

Intensive upfront therapy in newly-diagnosed multiple myeloma (MM) including induction therapy (IT), high-dose melphalan (MEL200), and autologous blood stem cell transplantation (ASCT) followed by consolidation and/or maintenance is mostly restricted to patients up to 65 years of age. Prospective phase III trial data in the era of novel agents for patients up to 70 years of age are not available. The GMMG-MM5 trial included 601 patients between 18 and 70 years of age, divided in three groups for the present analysis: ≤60 years (S1, *n* = 353), 61–65 years (S2, *n* = 107) and 66–70 years (S3, *n* = 141). Treatment consisted of a bortezomib-containing IT, MEL200/ASCT, consolidation, and maintenance with lenalidomide. Adherence to treatment was similar among patients of the three age groups. Overall toxicity during all treatment phases was increased in S2 and S3 compared to S1 (any adverse event/any serious adverse event: S1:81.7/41.8% vs. S2:90.7/56.5% vs. S3:87.2/68.1%, *p* = 0.05/<0.001). With respect to progression-free survival (log-rank *p* = 0.73), overall survival (log-rank *p* = 0.54) as well as time-to-progression (Gray’s *p* = 0.83) and non-relapse mortality (Gray’s *p* = 0.25), no differences were found between the three age groups. Our results imply that an intensive upfront therapy with a bortezomib-containing IT, MEL200/ASCT, lenalidomide consolidation, and maintenance should be applied to transplant-eligible MM patients up to 70 years of age.

## Introduction

Three-drug induction therapy (IT) including at least one novel agent, immunomodulatory agent (IMiD) and/or proteasome inhibitor (PI) and/or monoclonal antibody (moAb), followed by high-dose melphalan (at a dose of 200 mg/m^2^, MEL200)/autologous blood stem cell transplantation (ASCT) and lenalidomide maintenance until disease progression, is the frontline standard of care for newly-diagnosed multiple myeloma (MM) [1–4]. Most studies, however, investigating intense triplet/quadruplet IT, MEL200/ASCT, consolidation and/or maintenance therapy concepts, include only patients up to the age of 65 years [1, 5–7]. Only a minority of studies included patients aged up to 75 years, such as some of the total-therapy program trials [8]. Other studies in patients >65 years of age examined dose-reduced conditioning regimens (e.g., melphalan at a dose of 100 or 140 mg/m^2^, MEL100/140) along withASCT approaches and omitted IT [9, 10].

Treatment of newly-diagnosed MM nowadays involves triplet or quadruplet regimens including a moAb, a PI, an alkylating drug, and corticosteroids [11] as well as continuous therapy with IMiDs and corticosteroids [12–14], if patients are not considered transplant-eligible or >65 years of age. Further, novel agent-based therapy improved tolerability and decreased toxicity [15]. Therefore, age as a criterion for an intense upfront therapy sequence including IT, MEL200/ASCT, consolidation, and maintenance therapy needs to be reconsidered.

The German-Speaking Myeloma Multicenter Group (GMMG)-MM5 is a prospective, multicenter phase III trial applying bortezomib-based IT, upfront MEL200/ASCT, and lenalidomide consolidation as well as maintenance therapy in patients between 18 and 70 years of age. We addressed the question whether this intense therapeutic concept has similar toxicity, efficacy, and survival outcomes dependent on patient age. Thus, we conducted an analysis with focus on progression-free survival (PFS), overall survival (OS), time-to-progression (TTP), non-relapse mortality (NRM), response rates and toxicities with regard to patient age at randomization: ≤60 years (subgroup S1), 61–65 years (subgroup S2) and 66–70 years (subgroup S3).

## Patients and methods

### Study design and treatment

In the prospective, open-label, multicenter phase III trial GMMG-MM5 (EudraCT No. 2010-019173-16), 31 transplant and 74 associated sites throughout Germany participated. Results on the first and second primary endpoint have been published previously including details on the trial design, randomization methods, and recruitment period [16, 17]. The trial was approved by ethics committees of the University of Heidelberg as well as all participating sites and was conducted according to the European Clinical Trial Directive (2005) and the Declaration of Helsinki. All patients gave written informed consent.

### Eligibility criteria, study design, treatment, and assessments

The GMMG-MM5 trial included transplant-eligible patients from 18 to 70 years of age with previously untreated, newly-diagnosed MM requiring systemic therapy [18] and WHO performance status (WHO-PS) 0–2 (or 3 if MM-related). Systemic light chain amyloidosis and peripheral neuropathy ≥2° (according to the National Cancer Institute Common Terminology Criteria for Adverse Events, NCI CTCAE, version 4.0), but not renal impairment or failure, were important exclusion criteria.

IT within the GMMG-MM5 trial consisted of three cycles of either bortezomib/doxorubicine/dexamethasone (PAd, study arms A1 + B1) or bortezomib/cyclophosphamide/dexamethasone (VCD, study arms A2 + B2) followed by stem cell mobilization/collection [16] (Supplementary Material 1). MEL200/ASCT was adjusted to renal function. In case of less than near complete response (<nCR) after first MEL200/ASCT, a tandem MEL200/ASCT was recommended independent of patient age. MEL200/ASCT was carried out according to local standardized GMMG protocol. After MEL200/ASCT, two cycles of lenalidomide consolidation (25 mg, day 1–21, repeat day 29) were administered. Subsequently, two different lenalidomide maintenance therapy strategies were applied: either lenalidomide continuously for 2 years (LEN-2Y, study arms A1 + A2) *or* continuous lenalidomide for 2 years only in patients not achieving a complete response (CR) before start of *or* during maintenance therapy (LEN-CR, study arms B1 + B2). Starting dose of lenalidomide maintenance therapy was 10 mg/day. After three months, lenalidomide dose could be increased up to 15 mg/day if tolerated.

Response within the trial was assessed according to the International Myeloma Working Group (IMWG) criteria [19], including nCR as described [16]. High-risk cytogenetics, defined as either deletion 17p13 (subclonal in >10%) and/or translocation t(4;14) and/or translocation t(14;16) and/or gain 1q21 (>3 copies) were determined by fluorescence in-situ hybridization (FISH) as described earlier [16, 17].

Adverse events (AEs) were documented according to the NCI CTCAE (version 4.0, only if ≥3°, and ≥2° for infections, cardiac disorders, neuropathy or thromboembolic events). Serious adverse events (SAE) were recorded independent of CTCAE grade. For the MEL200/ASCT period, only SAE were recorded. AE and SAE were analyzed applying the MedDRA terminology.

### Design of the current subgroup analysis

For the present analysis, an expanded intention-to-treat (ITT) cohort (*n* = 601) was examined. Data were analyzed with respect to three predefined age groups: ≤60 years (subgroup S1), 61–65 years (subgroup S2) and 66–70 years (subgroup S3). This analysis is an unscheduled, exploratory subgroup analysis. Data base closure for the present analysis was June 2017.

### Statistical design and analysis

PFS was defined as time from randomization to disease progression or death from any cause, whichever occurs first. OS was defined as time from randomization or from first relapse/progression until death from any cause. Survival distributions of PFS/OS were estimated by utilizing the Kaplan–Meier method. Survival curve comparisons among age groups were conducted by using log-rank tests, differences were characterized by corresponding hazard ratios (HR) along with 95% confidence intervals (CI) and were displayed as forest plots for specific subgroups. Additionally, likelihood ratio tests were carried out to test a possible interaction between the predefined subgroups and age groups [20]. Distributions of follow-up times were estimated by the reverse Kaplan–Meier method [21]. To adjust for other predefined covariates of interest on PFS/OS, multivariate Cox regression models were fitted. Competing risks analyses of the competing events of progressive disease (PD, cause 1) and particularly NRM defined as death without previous PD (cause 2) were conducted from the date of randomization and date of ASCT. Incidence and survival curves were estimated by the Aalen-Johansen method [22]. Proportional cause-specific (CS) hazards models were fitted on the competing risks. For testing the equality of cumulative incidence curves, Gray’s test was utilized [23].

All survival analyses were based on the expanded ITT population (*n* = 601). All safety analyses were based on the safety population consisting of all patients randomized that received at least one dose of trial medication (*n* = 598). Patients were analyzed as treated. Frequency distributions of baseline characteristics, response rates, toxicities, and trial medication among age subgroups were compared inferentially by Fisher’s exact test for categorical variables and by the Kruskal–Wallis test for continuous variables. No multiplicity adjustment was done for exploratory analyses. For estimated effects, 95% CI were computed. All reported *p*-values were two-sided and considered to be statistically significant if ≤0.05. The statistical analyses were performed using R version 3.5.1 (www.r-project.org) [24].

## Results

### Baseline characteristics and adherence to treatment

There were 353, 107, and 141 patients in defined age groups S1, S2, and S3, respectively. The baseline patients’ and treatment characteristics are presented in Table 1. Glomerular filtration rate (GFR) significantly declined with increasing patient age (median GFR, S1:103.9 ml/min vs. S2:81.9 ml/min vs. S3:75.6 ml/min, *p* < 0.001). Simultaneously, International Staging System stage III was more common among S2 and S3 vs. S1 (ISS stage III, S1:24.4% vs. S2:31.8% vs. S3:31.9%, *p* = 0.04) [25]. However, rate of patients with renal impairment (RI, serum creatinine ≥2 mg/dl) at study entry was similar among age groups S1, S2, and S3 (RI, S1:12.5% vs. S2:15.9% vs. S3:15.6%, *p* = 0.54) as were revised ISS stages [26] (*p* = 0.15). At least one concomitant disease/medical condition other than MM was recorded in S1:89.8% vs. S2:93.5% vs. S3:96.5% (*p* = 0.04) of patients, respectively. More than one previous/concomitant cardiac and/or vascular disorder was reported in 10.8% (S1) vs. 27.1% (S2) vs. 29.1% (S3, *p* < 0.001) of patients, respectively.

**Table 1:** Baseline patient and treatment characteristics.

Characteristics	S1 (*N* = 353)	S2 (*N* = 107)	S3 (*N* = 141)	*p*
	*n*/%	*n*/%	*n*/%	
Sex				
Male	205/58.1	64/59.8	83/58.9	0.94
Female	148/41.9	43/40.2	58/41.1	
Age in years				
Median (range)	54 (32–60)	63 (61–65)	68 (66–70)	–
WHO performance status				
0	162/45.9	44/41.1	46/32.6	0.22
1	156/44.2	53/49.5	76/53.9	
2	25/7.1	9/8.4	13/9.2	
3	5/1.4	1/0.9	4/2.8	
Unknown	5/1.4	0/0.0	2/1.4	
Heavy chain isotype				
IgG	209/59.2	65/60.8	90/63.8	0.53
IgA	77/21.8	17/15.9	29/20.6	
LCD	62/17.6	24/22.4	19/13.5	
IgD	5/1.4	1/0.9	3/2.1	
Light chain isotype				
Kappa	233/66.0	69/64.5	102/72.3	0.31
Lambda	120/34.0	38/35.5	39/27.7	
Calcium elevation (calcium > 2.65 mmol/l)				
Yes	45/12.8	17/15.9	18/12.8	0.68
Renal insufficiency (creatinine > 177 μmol/l)				
Yes	44/12.5	17/15.9	22/15.6	0.54
Anemia (Hb < 10 g/dl or 2 g/dl < normal)				
Yes	179/50.7	55/51.4	78/55.3	0.65
Bone disease (lytic lesions^a^)				
Yes	316/89.5	99/92.5	127/90.1	0.69
ISS stage				
I	145/41.1	44/41.1	41/29.1	**0.04**
II	122/34.6	29/27.1	55/39.0	
III	86/24.4	34/31.8	45/31.9	
Revised ISS stage				
I	85/27.4	33/35.1	30/22.7	0.15
II	184/59.4	46/48.9	87/65.9	
III	41/13.2	15/16.0	15/11.4	
Adverse cytogenetics				
del 17p13				
*done*	321	93	135	0.40
*positive (% of done)*	42/13.1	8/8.6	13/9.6	
t (4;14)				
*done*	316	94	134	0.10
*positive (% of done)*	39/12.3	6/6.4	9/6.7	
gain 1q21 (>3 copies)				
*done*	310	92	134	0.56
*positive (% of done)*	30/9.7	8/8.7	17/12.7	
t (14;16)				
*done*	306	93	131	0.88
*positive (% of done)*	10/3.3	2/2.1	3/2.3	
any^b^				
*done*	301	90	132	0.21
*positive (% of done)*	96/31.9	20/22.2	36/27.3	
LDH *(serum)*				
≤ULN	298/84.4	89/84.0	122/87.1	0.71
>ULN	55/15.6	17/16.0	18/12.9	
Glomerular filtration rate (serum, ml/min)				
Median (range)	104 (6–232)	82 (14–154)	76 (14–152)	**<0.001**
Any previous/concomitant disease				
Yes	317/89.8	100/93.5	136/96.5	**0.04**
Cardiac and vascular disorders				
0–1	315/89.2	78/72.9	100/70.9	**<0.001**
>1	38/10.8	29/27.1	41/29.1	
Induction therapy (assigned by randomization)				
PAd	174/49.3	46/43.0	79/56.0	0.11
VCD	179/50.7	61/57.0	62/44.0	
Maintenance therapy strategy (assigned by randomization)				
LEN-2Y	169/47.9	58/54.2	73/51.8	0.46
LEN-CR	184/52.1	49/45.8	68/48.2	

Bold *p* values depict a statistically significant result.

*ISS* International Staging System, *WHO* World Health Organization, *LDH* lactate dehydrogenase, *>ULN* greater than the upper level of normal range, *LCD* light chain disease, *IgG/A* immunoglobulin G/A, *hb* hemoglobin, *LEN* lenalidomide, *PAd* bortezomib/doxorubicine/dexamethasone induction therapy, *VCD* bortezomib, cyclophosphamide, dexamethasone induction therapy, *LEN-2Y (study arms A1* *+* *A2*) continuous lenalidomide maintenance for 2 years, *LEN-CR (study arms B1* *+* *B2)* lenalidomide maintenance for 2 years, if no complete response (CR) was achieved.

^a^or myeloma-related osteopenia/osteoporosis.

^b^at least one high-risk aberration, including del 17p13, t (4;14), t(14;16) or gain 1q21 > 3 copies. ISS and revised ISS were calculated according to Greipp et al. [25] and Palumbo et al. [26].

**Fig. 1:**
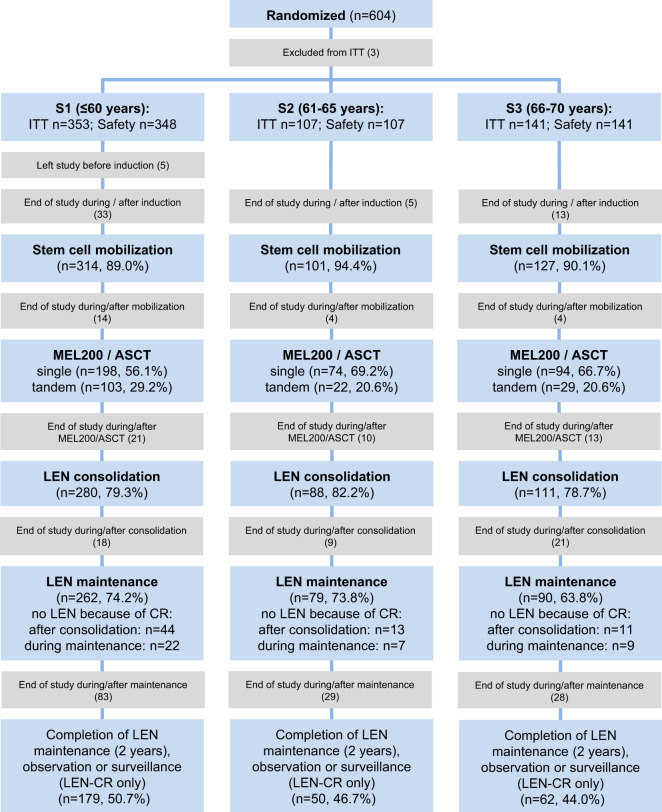
Consort diagram. Consort diagram is grouped by the three predefined age groups: ≤60 years (subgroup S1), 61–65 years (subgroup S2) and 66–70 years (subgroup S3). *n*/% numbers are from the intention-to-treat population of each age group. Light blue boxes indicate different trial sections or skipped therapy phases. Gray boxes summarize reasons for end of study between subsequent trial sections in the respective age groups S1, S2, and S3. For a detailed version of the consort diagram see Supplementary Fig. 1. ITT, intention-to-treat population; LEN, lenalidomide; MDS, myelodysplastic syndrome; MEL200, melphalan 200 mg/m^2^; ASCT, autologous blood stem cell transplantation; CR, complete response; AE, adverse event; PD, progressive disease.

As depicted in the consort diagram (Fig. 1, Supplementary Fig. 1), the proportions of ITT patients completing IT, receiving a first and second MEL200/ASCT, beginning lenalidomide consolidation and maintenance were similar among the three age groups. Tandem MEL200/ASCT was applied in 24.4 vs. 29.9% vs. 15.8% of ITT patients with unknown cytogenetic status, without or with adverse cytogenetics, respectively. At least 12 months of lenalidomide maintenance therapy were applied in 64.7% (S1) vs. 66.7% (S2) vs. 59.5% (S3) of patients (*p* = 0.61), respectively. Regular study completion was achieved in 50.7% vs. 46.7% vs. 44.0% of patients in age groups S1, S2, and S3. Consistently, median time to premature withdrawal from any cause within the study was similar between the three age groups (S1:14.1 vs. S2:18.0 vs. S3:13.6 months, log-rank *p* = 0.79, Supplementary Fig. 2).

### Response rates

Response rates, including progressive disease (PD), partial response/very good partial response or better (≥PR/≥ VGPR) and CR rates neither differed after IT nor post lenalidomide consolidation therapy (Supplementary Table 1). Best responses on study did not differ regarding ≥VGPR and CR but with respect to ≥PR rates (S1:94.7% vs. S2:98.1% vs. S3:89.8%, p = 0.02, Supplementary Table 1). Rates of ≥nCR after first MEL200/ASCT in patients without/with any adverse cytogenetic aberration were 41.7% vs. 57.5% (p = 0.003).

### Toxicities during study treatment, trial medication

AE and SAE during the subsequent study periods are depicted in Table 2 and Supplementary Table 2.

**Table 2:** Toxicities according to study periods: induction therapy, high-dose melphalan and maintenance therapy.

Events	S1 *n*/%	S2 *n*/%	S3 *n*/%	*p*
Induction therapy	*N* = 349	*N* = 108	*N* = 141	
Any AE/SAE	205/58.7	78/72.2	96/68.1	**0.02**
Infections and infestations (≥2°, SOC)	56/16.0	24/22.2	30/21.3	0.20
Blood and lymphatic system disorders (≥3°, SOC)	66/18.9	28/25.9	35/24.8	0.16
Gastrointestinal disorders (≥3°, SOC)	16/4.6	8/7.4	18/12.8	**0.008**
Cardiac disorders (≥2°, SOC)	4/1.1	3/2.8	3/2.1	0.40
Renal and urinary disorders (≥3°, SOC)	11/3.2	2/1.9	4/2.8	0.89
Neuropathy (≥2°, specific term)	25/7.2	9/8.3	8/5.7	0.70
Thromboembolic events (≥2°, specific term)	9/2.6	5/4.6	6/4.3	0.44
Leukocyto- and/or neutropenia (≥3°, specific term)	73/20.9	35/32.4	32/22.7	0.05
Thrombocytopenia (≥3°, specific term)	14/4.0	7/6.5	8/5.7	0.45
Anemia (≥3°, specific term)	15/4.3	6/5.6	7/5.0	0.80
Any SAE	74/21.2	41/38.0	57/40.4	**<0.001**
SAE due to infections and infestations (SOC)	20/5.7	16/14.8	14/9.9	**0.01**
First MEL200/ASCT	*N* = 302	*N* = 96	*N* = 123	
Any SAE	43/14.2	16/16.7	42/34.1	**<0.001**
SAE due to infections and infestations (SOC)	22/7.3	10/10.4	19/15.4	**0.04**
Second MEL200/ASCT	*N* = 104	*N* = 22	*N* = 29	
Any SAE	20/19.2	6/27.3	9/31.0	0.33
SAE due to infections and infestations (SOC)	11/10.6	4/18.2	3/10.3	0.52
Lenalidomide maintenance	*N* = 273	*N* = 87	*N* = 107	
Any AE/SAE	188/68.9	62/71.3	78/72.9	0.74
Infections and infestations (≥2°, SOC)	118/43.2	37/42.5	57/53.3	0.18
Blood and lymphatic system disorders (≥3°, SOC)	92/33.7	27/31.0	37/34.6	0.88
Gastrointestinal disorders (≥3°, SOC)	13/4.8	6/6.9	11/10.3	0.13
Cardiac disorders (≥2°, SOC)	1/0.4	0/0.0	3/2.8	0.07
Renal and urinary disorders (≥3°, SOC)	2/0.7	1/1.1	2/1.9	0.48
Neuropathy (≥2°, specific term)	12/4.4	3/3.4	4/3.7	1.0
Thromboembolic events (≥2°, specific term)	7/2.6	6/6.9	5/4.7	0.14
Leukocyto- and/or neutropenia (≥3°, specific term)	92/33.7	29/33.3	30/28.0	0.56
Thrombocytopenia (≥3°, specific term)	23/8.4	7/8.0	18/16.8	**0.05**
Anemia (≥3°, specific term)	5/1.8	2/2.3	2/1.9	0.90
Any SAE	81/29.7	30/34.5	50/46.7	**0.01**
SAE due to infections and infestations (SOC)	48/17.6	13/14.9	31/29.0	**0.02**

Detailed listing of (serious) adverse events according to treatment phases: induction therapy, MEL200/ASCT and maintenance therapy with respect to the three age groups ≤60 years (S1), 61–65 years (S2) and 66–70 years (S3). Adverse events were recorded applying the NCI CTCAE criteria (version 4.0, ≥2° for infections, cardiac disorders, neuropathy or thromboembolic events or if an serious adverse event occurred, otherwise, only if ≥3°) and systematically analyzed using the MedDRA terminology. For MEL200/ASCT, only SAE were available. Specific AE/SOC terms are presented if considered relevant and may subsummarize different primary terms according to MedDRA.

Bold *p* values are statistically significant.

*AE* adverse event, *SAE* serious AE, *NCI CTCAE* National Cancer Institute Common Terminology Criteria for Adverse Events, *SOC* System Organ Class (according to MedDRA terminology).

Overall toxicity during treatment phases (excluding SAE during MEL200/ASCT) was increased in S2/S3 vs. S1 (any AE/any SAE: S1:81.7/41.8% vs. S2:90.7/56.5% vs. S3:87.2/68.1%, *p* = 0.05/<0.001). Any AE/SAE was more frequent in S2/S3 vs. S1 during IT (S1:58.7% vs. S2:72.2% vs. S3:68.1%, *p* = 0.02) but not during lenalidomide maintenance therapy (S1:68.9% vs. S2:71.3% vs. S3:72.9%, *p* = 0.74). Rates of SAE were more frequent in the age groups S2/S3 vs. S1 during IT, and lenalidomide maintenance therapy (IT/lenalidomide maintenance: S1:21.2/29.7% vs. S2:38.0/34.5% vs. S3:40.4/46.7%, *p* < 0.001/0.01).

During first MEL200/ASCT, rates of SAE increased within age groups S1 to S3 (any SAE: S1:14.2% vs. S2:16.7% vs. S3:34.1%, *p* < 0.001). During second MEL200/ASCT, SAE rates between age groups S1 to S3 were increasing but not significantly different (any SAE: S1:19.2% vs. S2:27.3% vs. S3: 31.0%, *p* = 0.33).

Mortality from any cause within 100 days from last MEL200/ASCT (either single or tandem) was 1.9% (*n*/*N* = 10/520) in the overall cohort and higher in S2/S3 vs. S1 (S1:0.7% vs. S2:3.1% vs. S3:4.1%, *p* = 0.02).

The cumulative doses of the applied trial medication during IT, MEL200/ASCT, and lenalidomide maintenance can be found in Table 3. Mean dose of lenalidomide during maintenance therapy was lower in the age groups S2 and S3 vs. S1 (S1:11.5 vs. S2:10.6 vs. S3:10.6 mg/day, *p* < 0.001). The rates of dose reductions/discontinuations during induction and maintenance therapy were similar between the three predefined age groups (Supplementary Table 3).

**Table 3:** Trial medication during induction therapy, high-dose melphalan and lenalidomide maintenance therapy.

Medication mean (interquartile ranges)	S1	S2	S3	*p*
Induction therapy				
Bortezomib (cumulative mg/m^2^)	15.1 (14.7–15.7)	15.0 (15.1–15.7)	14.8 (14.5–15.7)	0.56
Doxorubicine (cumulative mg/m^2^)	106.4 (105.7–109.0)	107.5 (106.7–109.6)	105.9 (104.9–108.7)	0.13
Cyclophosphamide (cumulative mg/m^2^)	2564.2 (2619.6–2704.9)	2600.8 (2608.7–2700.0)	2574.8 (2647.3–2702.3)	0.52
Dexamethasone (mg/cycle)	277.4 (240–320)	285.2 (240–320)	270.5 (240–320)	**<0.001**
MEL200/ASCT				
First ASCT (melphalan, mg/m^2^/cycle)	196.4 (200.0–200.0)	197.8 (200.0–200.0)	206.3 (200.0–200.0)	0.05
Second ASCT (melphalan, mg/m^2^/cycle)	195.9 (200.0–200.0)	195.5 (200.0–200.0)	178.0 (183.0–200.0)	**<0.001**
Maintenance therapy				
Lenalidomide (mg/day)	11.5 (10.0–15.0)	10.6 (10.0–15.0)	10.6 (10.0–15.0)	**<0.001**

Applied doses of trial medication with respect to the three age groups ≤60 years (S1), 61–65 years (S2) and 66–70 years (S3).

Bold *p* values are statistically significant.

*MEL200* melphalan 200 mg/m^2^; *ASCT* autologous blood stem cell transplantation.

### Progression-free and overall survival, time-to-progression, and non-relapse mortality

**Fig. 2:**
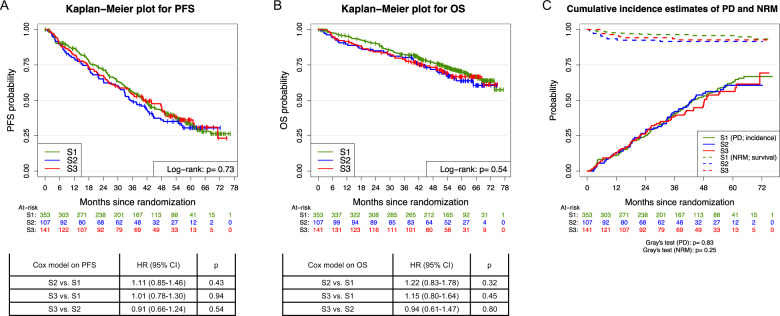
Progression-free, overall survival, time-to-progression, and non-relapse mortality from randomization. **a**, **b** Progression-free and overall survival (PFS, OS) from randomization with respect to the three age groups: ≤60 years (S1), 61–65 years (S2), and 66–70 years (S3) including univariate Cox models comparing single age groups. **c** Cumulative incidence estimates of competing events progressive disease (PD, cause 1) vs. non-relapse mortality (NRM, cause 2) from randomization shown as incidence and survival curves, respectively, for the age groups S1, S2 and S3. PFS, progression-free survival; OS, overall survival; TTP, time-to-progression; NRM, non-relapse mortality; HR, hazard ratio; 95% CI, 95% confidence interval.

In total, 366 PFS and 178 OS events were observed. Median follow-up time was 57.1 months (95% CI:55.6–59.2 months) for PFS and 57.6 months (95% CI:56.4–59.2 months) for OS, respectively. Neither PFS (log-rank *p* = 0.73) nor OS (log-rank *p* = 0.54) from randomization differed significantly among age groups (Fig. 2a, b). Median PFS was 40.8 vs. 35.0 vs. 40.9 months among age groups S1, S2, and S3, respectively. To further dissect progression-free survival between age groups, a competing risks model from time of randomization was built including two endpoints: either progressive disease (PD = TTP, 328 events) or death without PD ( = NRM, 35 events). No differences between the three age groups were observed for TTP (Gray’s *p* = 0.83) or NRM (Gray’s *p* = 0.25, Fig. 2c).

### Overall survival and therapies from first relapse/progression

Neither OS from first relapse/progression (log-rank *p* = 0.47, Supplementary Fig. 3) nor applied therapies at first relapse/progression differed between the three age groups (*p* = 0.88, Supplementary Table 4).

### Subgroup analyses according to baseline variables and defined age groups

**Fig. 3:**
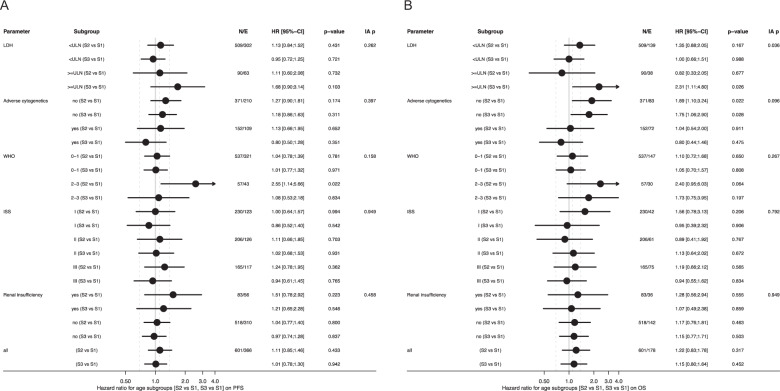
Forest plots on subgroup analyses for progression-free and overall survival from randomization. Forest plots on specific subgroups defined by baseline disease characteristics for **a** PFS and **b** OS from randomization. Age groups are defined as: ≤60 years (S1), 61–65 years (S2) and 66–70 years (S3). Renal insufficiency is defined as serum creatinine value of >177 μmol/l. Adverse cytogenetics were defined as at least one of the following aberrations: deletion17p13, translocation t(4;14), translocation t(14;16), gain 1q21 (>3 copies). PFS, progression-free survival; OS, overall survival; LDH, lactate dehydrogenase; ULN, upper limit of the normal; WHO, World Health Organization; IA p, interaction test *p* value.

Subgroups according to baseline variables with respect to a comparison of predefined age groups are shown in Fig. 3. Only patients from age group S2 with a WHO-PS of 2–3 had a worse PFS as compared to patients from age group S1 (HR = 2.55, *p* = 0.02), though the interaction-p (i-p) was not significant (WHO-PS 0–1 vs. 2–3, i-p = 0.16, Fig. 3a). OS for patients in age groups S2/S3 with standard-risk cytogenetics was shortened vs. S1 (S2/S3 vs. S1, HR = 1.89/1.75, *p* = 0.02/0.03; adverse cytogenetics no vs. yes, i-p = 0.10) as was OS from age group S3 vs. S1 in patients with LDH greater than the upper limit of the normal (LDH > ULN; S3 vs. S1, HR = 2.31, *p* = 0.03; LDH ≤ ULN vs. >ULN, i-p = 0.04, Fig. 3b).

### Multivariate analyses of progression-free and overall survival

Multivariate analyses on PFS and OS from randomization are displayed in Table 4. Adverse prognostic factors on PFS were: male sex (HR = 1.32, *p* = 0.02), ISS stages II/III (HR = 1.45/1.74, *p* = 0.009/<0.001), LDH > ULN (HR = 1.51, *p* = 0.01) and high-risk cytogenetics (HR = 1.77, *p* < 0.001). Age group S2 (HR = 1.61, *p* = 0.03), LEN-CR maintenance strategy (HR = 1.60, *p* = 0.005), WHO-PS 2–3 (HR = 1.95, *p* = 0.003), ISS stages II/III (HR = 1.76/2.66, *p* = 0.01/<0.001) and adverse cytogenetics (HR = 2.62, *p* < 0.001) were associated with a shortened OS.

**Table 4:** Multivariate models on progression-free and overall survival from randomization.

Factor	PFS		OS	
	HR (95% CI)	*p*	HR (95% CI)	*p*
Age group S2 (vs. S1)	1.28 (0.95–1.72)	0.11	1.61 (1.06–2.44)	**0.03**
Age group S3 (vs. S1)	1.00 (0.76–1.31)	0.99	1.20 (0.81–1.76)	0.36
Induction therapy (VCD)	0.88 (0.70–1.10)	0.27	1.06 (0.77–1.46)	0.73
Maintenance strategy (LEN-CR)	1.14 (0.91–1.43)	0.25	1.60 (1.15–2.23)	**0.005**
Sex (male)	1.32 (1.04–1.66)	**0.02**	1.16 (0.83–1.62)	0.38
WHO PS (>1)	1.37 (0.96–1.96)	0.08	1.95 (1.26–3.02)	**0.003**
ISS stage II	1.45 (1.10–1.91)	**0.009**	1.76 (1.13–2.72)	**0.01**
ISS stage III	1.74 (1.30–2.34)	**<0.001**	2.66 (1.70–4.16)	**<0.001**
LDH (>ULN)	1.51 (1.10–2.06)	**0.01**	1.32 (0.86–2.02)	0.21
Adverse cytogenetics (yes)	1.77 (1.39–2.25)	**<0.001**	2.62 (1.89–3.64)	**<0.001**
IgA subtype (yes)	1.02 (0.77–1.33)	0.91	1.07 (0.74–1.56)	0.71

Age groups are defined as: ≤60 years (S1), 61–65 years (S2) and 66–70 years (S3). Adverse cytogenetics were defined as at least one of the following aberrations: deletion17p13, translocation t(4;14), translocation t(14;16), gain 1q21 (>3 copies).

Bold *p* values are statistically significant.

*VCD* bortezomib, cyclophosphamide, dexamethasone, *LEN* lenalidomide, *CR* complete response, *WHO* World Health Organization, *PS* performance status, *ISS* International Staging System, *LDH* lactate dehydrogenase, *ULN* upper limit of normal, *Ig* immunoglobulin.

### Multivariate competing risk analyses on time-to-progression and non-relapse mortality

With respect to the assessment of potential differences for NRM and TTP from randomization (Fig. 2c) among the three age groups in the competing risks model, multivariate analyses adjusting for different variables with respect to TTP and NRM were performed (Table 5). TTP was adversely influenced by male sex (CSHR = 1.32, *p* = 0.03), ISS stages II/III (CSHR = 1.50/1.71, *p* = 0.006/<0.001), LDH > ULN (CSHR = 1.51, *p* = 0.02) and adverse cytogenetics (CSHR = 1.91, *p* < 0.001) whereas NRM was adversely influenced by WHO-PS > 1 only (CSHR = 3.49, *p* = 0.002) but not concomitant cardiac/vascular diseases (0–1 vs. >1; CSHR = 0.75, *p* = 0.56) nor age groups S2/S3 (CSHR = 2.20/1.67, *p* = 0.06/0.21). Similar results were observed in a competing risks model for TTP and NRM from ASCT (Supplementary Table 5).

**Table 5:** Multivariate models on time-to-progression and non-relapse mortality from randomization.

Factor	TTP		NRM	
	HR (95% CI)	*p*	HR (95% CI)	*p*
Age group S2 (vs. S1)	1.19 (0.86–1.63)	0.29	2.20 (0.95–5.08)	0.06
Age group S3 (vs. S1)	0.94 (0.70–1.25)	0.65	1.67 (0.74–3.76)	0.21
Induction therapy (VCD)	0.87 (0.68–1.10)	0.25	–	–
Maintenance strategy (LEN-CR)	1.15 (0.90–1.45)	0.26	–	–
Sex (male)	1.32 (1.03–1.69)	**0.03**	–	–
ISS stage II	1.50 (1.12–2.00)	**0.006**	–	–
ISS stage III	1.71 (1.25–2.34)	**<0.001**	–	–
LDH (>ULN)	1.51 (1.08–2.11)	**0.02**	–	–
Adverse cytogenetics (yes)	1.91 (1.48–2.46)	**<0.001**	–	–
IgA subtype (yes)	1.06 (0.80–1.41)	0.69	–	–
WHO PS (>1)	1.17 (0.78–1.74)	0.45	3.49 (1.56–7.80)	**0.002**
Cardiac/vascular disorders (>1)	–	–	0.75 (0.28–1.97)	0.56

Age groups are defined as: ≤60 years (S1), 61–65 years (S2) and 66–70 years (S3). Adverse cytogenetics were defined as at least one of the following aberrations: deletion17p13, translocation t(4;14), translocation t(14;16), gain 1q21 (>3 copies).

Bold *p* values are statistically significant.

*VCD* bortezomib, cyclophosphamide, dexamethasone, *LEN* lenalidomide, *CR* complete response, *WHO* World Health Organization, *PS* performance status, *ISS* International Staging System, *LDH* lactate dehydrogenase, *ULN* upper limit of normal, *Ig* immunoglobulin.

## Discussion

The present analysis of the GMMG-MM5 trial implies, that with an increasing patient age greater than 60 and up to 70 years (S2 and S3), more AE and SAE occur during treatment compared to patients ≤60 years (S1), but adherence to treatment and survival outcomes are similar (including PFS, OS, TTP, and NRM).

Beyond the comparison to patients ≤60 years (S1), there are no major differences regarding toxicities or survival between patients from 61 to 65 years (S2), which are routinely included in intense therapy trials applying MEL200/ASCT, and patients aged 66–70 years (S3) being mostly excluded from such trials. These findings imply that patients up to 70 years may be included in trials and treatment concepts applying an intense IT, MEL200/ASCT, and continued maintenance therapy to improve outcomes in the age group from 66 to 70 years. However, in this latter age group a direct, randomized comparison of modern conventional therapies and upfront MEL200/ASCT applying novel agents during IT and maintenance therapy is currently unavailable.

A single-arm phase II trial from the Italian Myeloma Group GIMEMA (EudraCT no. 2005-004714-32) had a similar study design compared to the GMMG-MM5 trial and included patients between 65 and 75 years or younger but ineligible for MEL200/ASCT [9]. In detail, the GIMEMA and GMMG trials are difficult to compare, because PAD IT and lenalidomide consolidation were applied for four cycles each and lenalidomide maintenance was given until PD in the GIMEMA study whereas the GMMG-MM5 trial applied only three IT and two consolidation cycles and lenalidomide maintenance was given for a fixed duration of 2 years or until achievement of a CR. Beyond this, melphalan was administered at a dose of 100 mg/m^2^ (MEL100) followed by ASCT and repeated thereafter in the GIMEMA study, whereas our study applied a single MEL200/ASCT and tandem MEL200/ASCT (if <nCR). Nonetheless, the GIMEMA trial and our present analysis support the hypothesis that patients >65 years tolerate intensive upfront therapeutic approaches and achieve deep and durable responses.

The moAb daratumumab significantly improved deep responses/rates of minimal residual disease negativity and PFS in transplant-ineligible patients as compared to standard of care [11, 12]. Whether patients aged >65 to 70 years benefit from either non-transplant daratumumab-based triplet/quadruplet therapies or IT, upfront ASCT, consolidation, and maintenance remains an open question. Presented follow-up times of the ALCYONE (NCT02195479) and MAIA (NCT02252172) are short (16.5/28.0 months) with median PFS results not reached yet [11, 12] and large proportions of patients ≥70 years of age (e.g., 79%) impair a direct comparison to our results. It has to be considered that patients >65 years of age, if transplant-eligible, might not be able to receive MEL200/ASCT at first or later disease relapse/progression, especially with regard to long first line PFS in the era of novel agent combinations, e.g., with bortezomib/lenalidomide/dexamethasone (median PFS of 65 months) [27]. Results from the CASSIOPEIA (NCT02541383) [7], GRIFFIN (NCT02874742, including patients up to 70 years of age) [28] and the ongoing PERSEUS (NCT03710603) trial as well as the favorable safety profile of daratumumab in quadruplet IT, consolidation and lenalidomide maintenance therapy will likely result in an approval of daratumumab in the near future for transplant-eligible patients. Thus, making it an attractive option for transplant-candidates aged >65 to 70 years.

Rather than chronological age, geriatric assessment and performance status appear to be crucial to guide therapeutic intensity in newly-diagnosed MM. Previous studies, mainly performed in transplant-ineligible patients, demonstrated that toxicity, rates of treatment discontinuation and ultimately outcomes are inferior based on geriatric and performance assessments [29–32]. Our analyses revealed that WHO-PS (0–1 vs. >1) but not age group (S2/S3 vs. S1) had a statistically significant impact on both OS and NRM in multivariate models (OS: HR = 1.90, *p* = 0.004/NRM: CSHR = 3.49, *p* = 0.002). The adverse prognostic effect of WHO-PS as part of a simplified frailty assessment is supported by other trials: a recent subgroup analysis from the FIRST trial (NCT00689936) identified a dichotomized Eastern Cooperative Oncology Group (ECOG) score (0–1 vs. ≥2) alone to be a predictor for PFS, OS (frail vs. non-frail; PFS: HR = 1.36, *p* < 0.001; OS: HR = 1.86, *p* < 0.001) and time to treatment-discontinuation (frail vs. non-frail; HR = 1.66, *p* = 0.03) in newly-diagnosed, transplant-ineligible patients [32]. This is in line with registry-based analyses on ASCT, where e.g., Karnofsky PS had a significant impact on PFS/OS (<80 vs. 100; PFS/OS: HR = 1.59/1.64, p = 0.008/<0.001). Based on these findings and our present analysis, PS rather than chronological age should be one major selection criterion to consider upfront ASCT. Besides, comorbidities, response, adverse events and patients’ preferences should be taken into account. In our trial, eligibility for MEL200/ASCT was indirectly assessed prior to IT upon randomization. Though patients with renal failure (including hemodialysis) and mild cardiac disease (New York Heart Association [NYHA] Functional Classification grade I or II) were included, assessment of transplant-eligibility should be performed repeatedly and prior to ASCT. Detailed data on the importance of specific comorbidities (e.g., cardiac disorders) is desirable to further guide decisions on transplant-eligibility.

Infections remain a major cause for morbidity and mortality in MM patients [33, 34]. With an increasing age (S2/S3 vs. S1), AE and SAE due to infections were more frequent in our cohort, though this did not result in inferior survival outcomes or a shortened median time on study. Recent results from the randomized, double-blind phase III TEAMM trial (Eudra CT no. 2011-000366-35) show a reduced number of first febrile episode or death during the first four months in patients receiving levofloxacin (500 mg orally once daily) vs. placebo during the first four months of anti-MM treatment (levofloxacin vs. placebo: 19 vs. 27%; HR = 0.66, 95% CI: 0.51–0.86, *p* = 0.002) [35]. In particular, patients >65 years of age (HR = 0.62) and with a poor ECOG score of 2–4 (HR = 0.52) had a significant benefit from levofloxacin prophylaxis vs. placebo as per univariate analyses. In the GMMG-MM5 trial, antibiotic prophylaxis was mandatory for all patients during IT (using ciprofloxacin [500 mg] or cotrimoxazole [960 mg] twice daily). Whether antibiotic prophylaxis should be applied to all newly-diagnosed MM patients or risk-adapted, e.g. based on predictive scores for early treatment-emergent severe infections [36], remains an open question and warrants further randomized, controlled clinical trials.

Patients in the age group S2 but not S3 have a dismal OS in our multivariate analyses compared to S1 (S2 vs. S1: HR = 1.58, *p* = 0.03). A possible explanation for this observation and downside of randomized controlled trials is patient selection: older patients (age group S3) may be selected more carefully for trial inclusion whereas patients in the intermediate age group S2 were selected more liberal since this age group is routinely recruited in intensive therapy protocols (e.g., in previous GMMG trials) [37].

Based on our results we assume that first MEL200/ASCT may not be dose reduced in patients between 66 and 70 years of age (S3). In this particular age group, upfront application of MEL200/ASCT is a promising strategy, because patients in this subgroup may be too old and/or frail for a MEL200/ASCT at first or later disease relapse. Further, MEL200/ASCT is superior to MEL100/ASCT regarding PFS (HR = 0.69, *p* = 0.01) but not OS (HR = 0.74, *p* = 0.13) in a randomized phase III trial including patients up to 65 years of age (NCT00950768) [38] and considered the most appropriate, widely used conditioning regimen in MM [1–3, 39]. Retrospective registry and single center data [40–42] further support that MEL200/ASCT can be applied safely in patients aged >65 years with similar survival outcomes (e.g., 60–69 vs. 18–59 years of age: 3-year PFS: 38 vs. 42%, *p* = 0.28 and 3-year NRM: 3 vs. 2%, *p* = 0.39). However, other trials including patients aged >65 years demonstrated that a dose reduced ASCT (e.g., MEL100 or MEL140) is feasible with a low toxicity profile [9, 10] and may be considered as a valid alternative in patients with relevant comorbidities and up to 70 years of age.

Whether upfront tandem MEL200/ASCT should be performed in patients >65 years of age is a matter of debate. Registry data demonstrate that similar proportions of patients aged 65–69 years compared to younger patients (e.g., 60–64 years of age) received a tandem ASCT in the period from 2006 to 2010 (15.9% vs. 14.0%). However, this analysis did not report on the conditioning regimens used, nor dosages [41]. Randomized phase III trials on single vs. tandem ASCT approaches conducted prior to the era of novel agents observed a response-dependent benefit of tandem ASCT in patients not achieving ≥VGPR after first ASCT [43, 44]. The applied response-adapted tandem MEL200/ASCT policy (if <nCR) in our present study was based on these previous findings when the trial was initiated in 2009 and is controversial nowadays. Of note, in our analysis patients with adverse cytogenetics more frequently achieved ≥nCR after first MEL200/ASCT compared to standard risk and thus missed a second, response-adapted MEL200/ASCT more likely. Further, withdrawal (in case of CR) vs. continuation of lenalidomide maintenance in the present GMMG-MM5 trial had a significant impact on the prognosis of adverse cytogenetics as described earlier [17]. Taken together, a second MEL200/ASCT should be used with caution, especially in patients >65 years of age (S3). The significantly lower applied MEL dose during second ASCT in the age group S3 vs. S2/S1 as well as lower numbers of patients undergoing a second MEL200/ASCT in the age groups S3/S2 vs. S1 further support this notion.

Recent findings from the European Myeloma Network (EMN) 02 (NCT01208766) trial [45] demonstrated a significantly prolonged 5-year PFS/OS in the tandem ASCT group (vs. single ASCT; PFS/OS: HR = 0.74/0.62, *p* = 0.036/0.022). The benefit of tandem ASCT was most pronounced in patients with adverse cytogenetics. In contrast, the STAMINA trial from the Blood and Marrow Transplant Clinical Trials Network (BMT CTN; NCT01109004) [46] did not observe any difference in 38-month PFS/OS for single vs. tandem ASCT (PFS/OS: 53.9/83.7% vs. 58.5%/81.8%). Based on these findings, upfront tandem ASCT should be carefully evaluated on an individual basis, including adverse disease characteristics/cytogenetics, prior response to and severe side effects during IT/first ASCT, comorbidities, and patient preferences.

Recent phase III trials in transplant-ineligible MM demonstrated the feasibility of continued therapy in patients >65 years of age, applying continuous lenalidomide therapy, either in combination with dexamethasone and/or daratumumab [12–14]. Our current results demonstrate similar toxicities, rates of dose reductions/therapy discontinuations and adherence to lenalidomide maintenance treatment in the three age groups. Thus, lenalidomide maintenance therapy at a starting dose of 10 mg/day in patients >65 years of age after upfront ASCT appears practicable.

Limitations of our study include suboptimal IT, single agent consolidation and fixed duration/response-adapted maintenance therapy. Nowadays a triplet or quadruplet IT combining an IMiD and PI with dexamethasone plus a moAb (e.g., daratumumab) is considered appropriate, and four to six IT cycles should be applied [7, 28, 47]. In addition, after ASCT consolidation with the IT regimen is widely used [1, 7, 28, 45, 47]. Rather than a fixed duration maintenance therapy, continuous therapy until disease progression or unacceptable toxicity is standard of care [4]. Lastly, the present analysis is exploratory and was not preplanned, thus sample size regarding this analysis was not determined prior to trial initiation.

Taken together, our present analysis demonstrates that an intense treatment approach, including IT, MEL200/ASCT, consolidation, and maintenance therapy can be applied in patients up to 70 years of age if they are considered transplant-eligible. This should be considered in clinical routine and design of further clinical trials evaluating intense therapeutic concepts.

## Supplementary information

Supplementary Figures and Tables

Supplemental material 1

Supplemental material 2
