# Publisher Correction: Bortezomib-based induction, high-dose melphalan and lenalidomide maintenance in myeloma up to 70 years of age

**DOI:** 10.1038/s41375-021-01357-4

**Published:** 2021-11-16

**Authors:** Elias K. Mai, Kaya Miah, Uta Bertsch, Jan Dürig, Christof Scheid, Katja C. Weisel, Christina Kunz, Markus Munder, Hans-Walter Lindemann, Maximilian Merz, Dirk Hose, Anna Jauch, Anja Seckinger, Steffen Luntz, Sandra Sauer, Stephan Fuhrmann, Peter Brossart, Ahmet Elmaagacli, Martin Goerner, Helga Bernhard, Martin Hoffmann, Marc S. Raab, Igor W. Blau, Mathias Hänel, Axel Benner, Hans J. Salwender, Hartmut Goldschmidt, for the German-speaking Myeloma Multicenter Group (GMMG)

**Affiliations:** 1 Department of Internal Medicine V, University Hospital Heidelberg, Heidelberg, Germany; 2 National Center for Tumor Diseases (NCT) Heidelberg, Heidelberg, Germany; 3 Division of Biostatistics, German Cancer Research Center (DKFZ), Heidelberg, Germany; 4 Department of Hematology, University Clinic Essen, Essen, Germany; 5 Department of Internal Medicine I, University Hospital Cologne, Cologne, Germany; 6 Department of Oncology, Hematology and Bone Marrow Transplantation with Section of Pneumology, University Medical Center Hamburg-Eppendorf, Hamburg, Germany; 7 Department of Hematology, Oncology and Immunology, University Hospital Tübingen, Tübingen, Germany; 8 Institute of Child Nutrition, Max Rubner Institute, Federal Research Institute of Nutrition and Food, Karlsruhe, Germany; 9 Department of Internal Medicine III, University Medical Center Mainz, Mainz, Germany; 10 Department of Hematology and Oncology, Katholisches Krankenhaus Hagen, Hagen, Germany; 11 Institute of Human Genetics, University of Heidelberg, Heidelberg, Germany; 12 Coordination Centre for Clinical Trials, University Hospital Heidelberg, Heidelberg, Germany; 13 Department of Hematology and Oncology, Helios Hospital Berlin Buch, Berlin, Germany; 14 University Hospital Bonn, Bonn, Germany; 15 Department of Hematology and Oncology, Asklepios Hospital Hamburg St. Georg, Hamburg, Germany; 16 Department of Hematology, Oncology and Palliative Care, Klinikum Bielefeld, Bielefeld, Germany; 17 Department of Internal Medicine V, Klinikum Darmstadt, Darmstadt, Germany; 18 Medical Clinic A, Klinikum Ludwigshafen, Ludwigshafen, Germany; 19 Medical Clinic, Charité University Medicine Berlin, Berlin, Germany; 20 Department of Internal Medicine III, Klinikum Chemnitz, Chemnitz, Germany; 21 Department of Hematology and Oncology, Asklepios Hospital Hamburg Altona, Hamburg, Germany

**Keywords:** Myeloma, Randomized controlled trials, Stem-cell research

Correction to: *Leukemia* 10.1038/s41375-020-0976-9

The article Bortezomib-based induction, high-dose melphalan and lenalidomide maintenance in myeloma up to 70 years of age, written by Elias K. Mai, Kaya Miah, Uta Bertsch, Jan Dürig, Christof Scheid, Katja C. Weisel, Christina Kunz, Markus Munder, Hans-Walter Lindemann, Maximilian Merz, Dirk Hose, Anna Jauch, Anja Seckinger, Steffen Luntz, Sandra Sauer, Stephan Fuhrmann, Peter Brossart, Ahmet Elmaagacli, Martin Goerner, Helga Bernhard, Martin Hoffmann, Marc S. Raab, Igor W. Blau, Mathias Hänel, Axel Benner, Hans J. Salwender, and Hartmut Goldschmidt for the German-speaking Myeloma Multicenter Group (GMMG), was originally published Online First without Open Access. After publication in volume 35, pages 809–822, the author decided to opt for Open Choice and to make the article an Open Access publication. Therefore, the copyright of the article has been changed to © The Author(s) 2020 and the article is forthwith distributed under the terms of the Creative Commons Attribution.

