# Supplementary material for: Bortezomib-based induction, high-dose melphalan and lenalidomide maintenance in myeloma up to 70 years of age
Source: Leukemia. 2020 Jul 20;35(3):809–22. doi: 10.1038/s41375-020-0976-9 (PMC8318883; doi:10.1038/s41375-020-0976-9)
Supplement: Supplementary file 1 — Supplementary Figures and Tables [file 41375_2020_976_MOESM1_ESM.pdf]

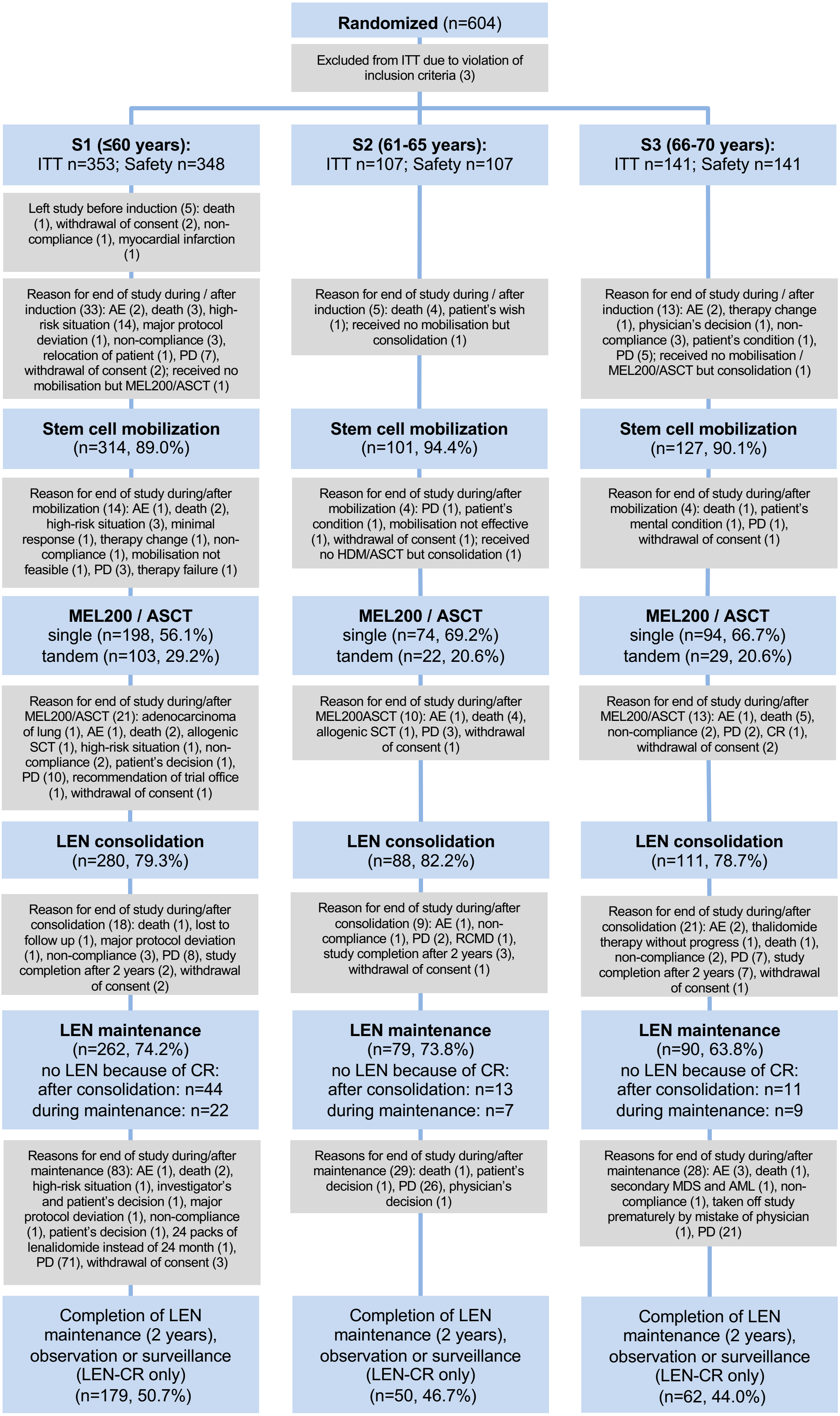
Randomized (n=604)
Excluded from ITT due to violation of inclusion criteria (3)
S1 (≤60 years): ITT n=353; Safety n=348
S2 (61-65 years): ITT n=107; Safety n=107
S3 (66-70 years): ITT n=141; Safety n=141
Left study before induction (5): death (1), withdrawal of consent (2), non-compliance (1), myocardial infarction (1)
Reason for end of study during / after induction (33): AE (2), death (3), high-risk situation (14), major protocol deviation (1), non-compliance (3), relocation of patient (1), PD (7), withdrawal of consent (2); received no mobilisation but MEL200/ASCT (1)
Reason for end of study during / after induction (5): death (4), patient's wish (1); received no mobilisation but consolidation (1)
Reason for end of study during / after induction (13): AE (2), therapy change (1), physician's decision (1), non-compliance (3), patient's condition (1), PD (5); received no mobilisation / MEL200/ASCT but consolidation (1)
Stem cell mobilization (n=314, 89.0%)
Stem cell mobilization (n=101, 94.4%)
Stem cell mobilization (n=127, 90.1%)
Reason for end of study during/after mobilization (14): AE (1), death (2), high-risk situation (3), minimal response (1), therapy change (1), non-compliance (1), mobilisation not feasible (1), PD (3), therapy failure (1)
Reason for end of study during/after mobilization (4): PD (1), patient's condition (1), mobilisation not effective (1), withdrawal of consent (1); received no HDM/ASCT but consolidation (1)
Reason for end of study during/after mobilization (4): death (1), patient's mental condition (1), PD (1), withdrawal of consent (1)
MEL200 / ASCT single (n=198, 56.1%) tandem (n=103, 29.2%)
MEL200 / ASCT single (n=74, 69.2%) tandem (n=22, 20.6%)
MEL200 / ASCT single (n=94, 66.7%) tandem (n=29, 20.6%)
Reason for end of study during/after MEL200/ASCT (21): adenocarcinoma of lung (1), AE (1), death (2), allogenic SCT (1), high-risk situation (1), non-compliance (2), patient's decision (1), PD (10), recommendation of trial office (1), withdrawal of consent (1)
Reason for end of study during/after MEL200ASCT (10): AE (1), death (4), allogenic SCT (1), PD (3), withdrawal of consent (1)
Reason for end of study during/after MEL200/ASCT (13): AE (1), death (5), non-compliance (2), PD (2), CR (1), withdrawal of consent (2)
LEN consolidation (n=280, 79.3%)
LEN consolidation (n=88, 82.2%)
LEN consolidation (n=111, 78.7%)
Reason for end of study during/after consolidation (18): death (1), lost to follow up (1), major protocol deviation (1), non-compliance (3), PD (8), study completion after 2 years (2), withdrawal of consent (2)
Reason for end of study during/after consolidation (9): AE (1), non-compliance (1), PD (2), RCMD (1), study completion after 2 years (3), withdrawal of consent (1)
Reason for end of study during/after consolidation (21): AE (2), thalidomide therapy without progress (1), death (1), non-compliance (2), PD (7), study completion after 2 years (7), withdrawal of consent (1)
LEN maintenance (n=262, 74.2%) no LEN because of CR: after consolidation: n=44 during maintenance: n=22
LEN maintenance (n=79, 73.8%) no LEN because of CR: after consolidation: n=13 during maintenance: n=7
LEN maintenance (n=90, 63.8%) no LEN because of CR: after consolidation: n=11 during maintenance: n=9
Reasons for end of study during/after maintenance (83): AE (1), death (2), high-risk situation (1), investigator's and patient's decision (1), major protocol deviation (1), non-compliance (1), patient's decision (1), 24 packs of lenalidomide instead of 24 month (1), PD (71), withdrawal of consent (3)
Reasons for end of study during/after maintenance (29): death (1), patient's decision (1), PD (26), physician's decision (1)
Reasons for end of study during/after maintenance (28): AE (3), death (1), secondary MDS and AML (1), non-compliance (1), taken off study prematurely by mistake of physician (1), PD (21)
Completion of LEN maintenance (2 years), observation or surveillance (LEN-CR only) (n=179, 50.7%)
Completion of LEN maintenance (2 years), observation or surveillance (LEN-CR only) (n=50, 46.7%)
Completion of LEN maintenance (2 years), observation or surveillance (LEN-CR only) (n=62, 44.0%)

Supplemental Figure 1: Detailed consort diagram

Consort diagram is grouped by the three predefined age groups: ≤60 years (subgroup S1), 61-65 years (subgroup S2) and 66-70 years (subgroup S3). n/% numbers are from the intention-to-treat population of each age group. Light blue boxes indicate different trial sections or skipped therapy phases. Grey boxes summarize reasons for end of study between subsequent trial sections in the respective age groups S1, S2 and S3.

Abbreviations: ITT, intention-to-treat population; LEN, lenalidomide; MDS, myelodysplastic syndrome; MEL200, melphalan 200mg/m2; ASCT, autologous blood stem cell transplantation; CR, complete response; AE, adverse event; PD, progressive disease.

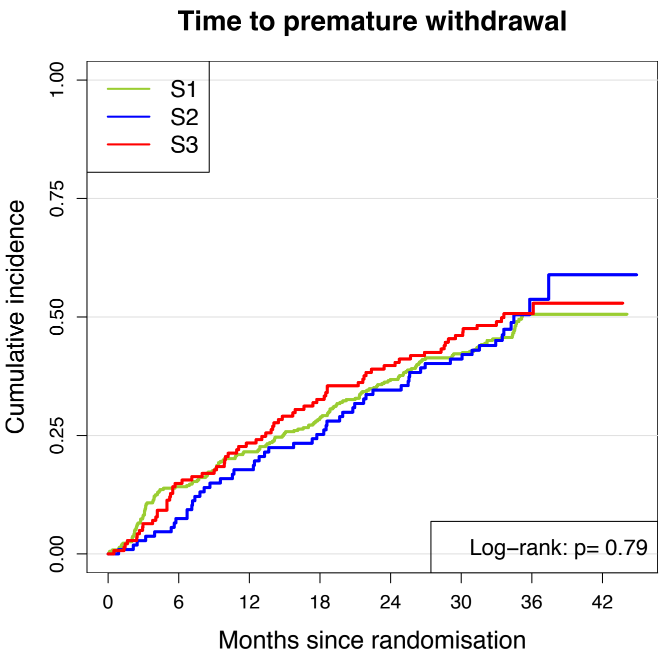

Subgroup	Median time to premature study withdrawal (months, minimum-maximum)
S1	14.1 (0.1-35.2)
S2	18.0 (0.9-37.4)
S3	13.6 (0.5-36.1)

Supplemental Figure 2: Time to premature study withdrawal

Distribution of time to premature withdrawal from the trial and corresponding median time with respect to the three age groups ≤60 years (S1), 61-65 years (S2) and 66-70 years (S3).

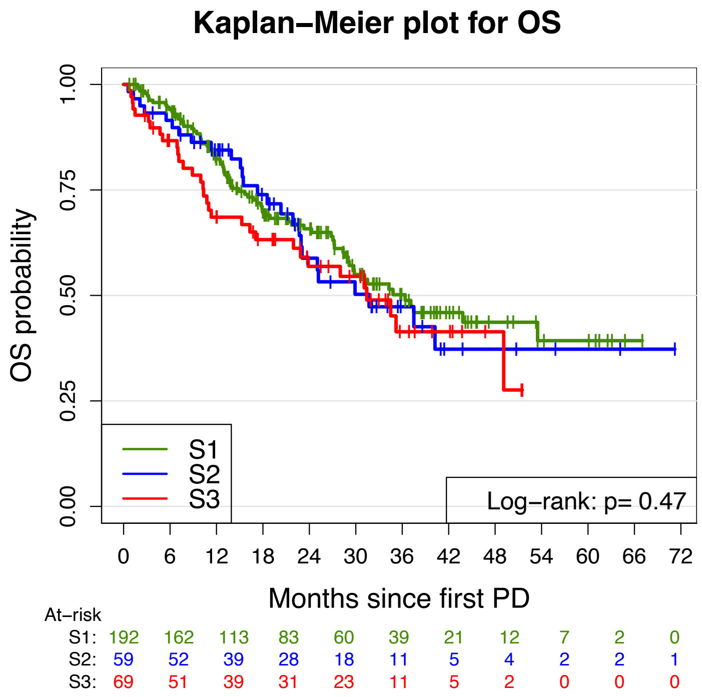

Cox model on OS	HR (95% CI)	p
S2 vs. S1	1.10 (0.70-1.71)	0.69
S3 vs. S1	1.30 (0.86-1.96)	0.22
S3 vs. S2	1.18 (0.70-1.98)	0.53

Supplemental Figure 3: Overall survival from first relapse/progression

Overall survival (OS) from first relapse/progression with respect to the three age groups: ≤60 years (S1), 61-65 years (S2) and 66-70 years (S3) including univariate Cox models comparing age groups.

Abbreviations: OS, overall survival; PD, progressive disease; HR, hazard ratio; 95% CI, 95% confidence interval.

Response rates (n / %)	S1	S2	S3	p
Post induction therapy	N=335	N=104	N=132	
PD	11 / 3.3	1 / 1.0	5 / 3.8	0.39
≥ PR	259 / 77.3	89 / 85.6	98 / 74.2	0.10
≥ VGPR	122 / 36.4	43 / 41.4	43 / 32.6	0.38
CR	19 / 5.7	6 / 5.8	5 / 3.8	0.71
Post consolidation therapy	N=277	N=85	N=109	
PD	3 / 1.1	1 / 1.2	1 / 0.9	1.0
≥ PR	264 / 95.3	84 / 98.8	105 / 96.3	0.40
≥ VGPR	210 / 75.8	72 / 84.7	87 / 79.8	0.20
CR	78 / 28.2	29 / 34.1	27 / 24.8	0.37
Best response on study	N=338	N=105	N=137	
≥ PR	320 / 94.7	103 / 98.1	123 / 89.8	**0.02**
≥ VGPR	269 / 79.6	87 / 82.9	109 / 79.6	0.76
CR	130 / 38.5	41 / 39.0	49 / 35.8	0.85

Supplemental Table 1: Response rates post induction therapy, post lenalidomide consolidation therapy and best response on study

Response rates according to Durie et al. (2006, Leukemia) with respect to the three age groups ≤60 years (S1), 61-65 years (S2) and 66-70 years (S3).

Bold p values are statistically significant.

Abbreviations: PD, progressive disease; ≥PR, partial response or better; ≥VGPR, very good partial response or better; CR, complete response.

Supplemental Table 2: Toxicities (only ≥3 grade) according to study periods: induction therapy, high dose melphalan and maintenance therapy

Detailed listing of (serious) adverse events according to treatment phases: induction therapy, HDM/ASCT and maintenance therapy with respect to the three age groups ≤60 years (S1), 61-65 years (S2) and 66-70 years (S3). Adverse events were recorded applying the NCI CTCAE criteria (version 4.0, ≥3° or if an serious adverse event occurred) and systematically analysed using the MedDRA terminology. For MEL200/ASCT, only SAE were available. Specific AE / SOC terms are presented if considered relevant and may sub summarize different primary terms according to MedDRA. Bold p values are statistically significant. Abbreviations: AE, adverse event; SAE, serious AE; NCI CTCAE, National Cancer Institute Common Terminology Criteria for Adverse Events; SOC, System Organ Class (according to MedDRA terminology).

Events	S1<br>n / %	S2<br>n / %	S3<br>n / %	p
Induction therapy	N=349	N=108	N=141	
Any AE/SAE (only ≥3°)	169 / 48.4	71 / 65.7	84 / 59.6	**0.002**
Infections and infestations (≥ 3°, SOC)	23 / 6.6	16 / 14.8	17 / 12.1	**0.02**
Blood and lympathic system disorders (≥ 3°, SOC)	66 / 18.9	28 / 25.9	35 / 24.8	0.16
Gastrointestinal disorders (≥ 3°, SOC)	16 / 4.6	8 / 7.4	18 / 12.8	**0.008**
Cardiac disorders (≥ 3°, SOC)	4 / 1.1	3 / 2.8	3 / 2.1	0.40
Renal and urinary disorders (≥ 3°, SOC)	11 / 3.2	2 / 1.9	4 / 2.8	0.89
Neuropathy (≥ 3°, specific term)	3 / 0.9	2 / 1.9	1 / 0.7	0.53
Thromboembolic events (≥ 3°, specific term)	6 / 1.7	2 / 1.9	1 / 0.7	0.72
Leukocyto- and/or Neutropenia (≥ 3°, specific term)	73 / 20.9	35 / 32.4	32 / 22.7	0.05
Thrombocytopenia (≥ 3°, specific term)	14 / 4.0	7 / 6.5	8 / 5.7	0.54
Anemia (≥ 3°, specific term)	15 / 4.3	6 / 5.6	7 / 5.0	0.80
Any SAE	74 / 21.2	41 / 38.0	57 / 40.4	**<0.001**
SAE due to Infections and infestations (SOC)	20 / 5.7	16 / 14.8	14 / 9.9	**0.01**
First MEL200/ASCT	N=302	N=96	N=123	
Any SAE	43 / 14.2	16 / 16.7	42 / 34.1	**<0.001**
SAE due to Infections and infestations (SOC)	22 / 7.3	10 / 10.4	19 / 15.4	**0.04**
Second MEL200/ASCT	N=104	N=22	N=29	
Any SAE	20 / 19.2	6 / 27.3	9 / 31.0	0.33
SAE due to Infections and infestations (SOC)	11 / 10.6	4 / 18.2	3 / 10.3	0.52
Lenalidomide maintenance	N=273	N=87	N=107	
Any AE/SAE (only ≥3°)	159 / 58.2	53 / 60.9	70 / 65.4	0.45
Infections and infestations (≥ 3°, SOC)	54 / 19.8	14 / 16.1	34 / 31.8	**0.02**
Blood and lympathic system disorders (≥ 3°, SOC)	92 / 33.7	27 / 31.0	37 / 34.6	0.88
Gastrointestinal disorders (≥ 3°, SOC)	13 / 4.8	6 / 6.9	11 / 10.3	0.13
Cardiac disorders (≥ 3°, SOC)	1 / 0.4	0 / 0.0	3 / 2.8	0.07
Renal and urinary disorders (≥ 3°, SOC)	2 / 0.7	1 / 1.1	2 / 1.9	0.48
Neuropathy (≥ 3°, specific term)	3 / 1.1	1 / 1.1	1 / 0.9	1.0
Thromboembolic events (≥ 3°, specific term)	2 / 0.7	3 / 3.4	5 / 4.7	**0.02**
Leukocyto- and/or Neutropenia (≥ 3°, specific term)	92 / 33.7	29 / 33.3	30 / 28.0	0.56
Thrombocytopenia (≥ 3°, specific term)	23 / 8.4	7 / 8.0	18 / 16.8	**0.05**
Anemia (≥ 3°, specific term)	5 / 1.8	2 / 2.3	2 / 1.9	0.90
Any SAE	81 / 29.7	30 / 34.5	50 / 46.7	**0.01**
SAE due to Infections and infestations (SOC)	48 / 17.6	13 / 14.9	31 / 29.0	**0.02**

Dose modifications in %	S1	S2	S3	p
Induction therapy				
**Bortezomib**				
full dose	77.6	76.2	76.4	0.43
reduced dose	5.2	5.3	5.1	
dose interrrupted and resumed	1.7	1.2	1.4	
discontinuation of treatment	1.2	0.6	2.9	
other	14.4	16.6	14.2	
**Doxorubicine**				
full dose	91.6	91.2	91.8	0.53
reduced dose	1.8	0.0	1.3	
dose interrrupted and resumed	0.4	1.5	0.0	
discontinuation of treatment	0.4	0.7	0.4	
other	5.8	6.6	6.5	
**Cyclophosphamide**				
full dose	85.9	89.6	87.0	0.68
reduced dose	9.3	8.7	10.9	
dose interrrupted and resumed	0.4	0.0	0.0	
discontinuation of treatment	0.2	0.0	0.0	
other	4.2	1.6	2.2	
**Dexamethasone**				
full dose	83.0	81.5	83.6	0.65
reduced dose	2.3	1.6	3.4	
dose interrrupted and resumed	1.2	1.2	1.2	
discontinuation of treatment	1.0	0.6	1.2	
other	12.5	15.1	10.6	
Maintenance therapy				
**Lenalidomide**				
reduced dose	5.6	7.5	4.0	0.93
dose interrrupted and resumed	75.4	76.3	76.8	
discontinuation of treatment	15.3	11.8	15.2	
other	3.7	4.3	4.0	

Supplemental Table 3: Rates of dose reductions and discontinuations during induction and lenalidomide maintenance therapy

Summary of dose modifications across all cycles of induction therapy and lenalidomide maintenance in the three age groups: ≤60 years (S1), 61-65 years (S2) and 66-70 years (S3). Bold p values are statistically significant. Numbers are percent (%).

Abbreviations: none.

Therapies at first disease relapse /progression (n / %)	S1	S2	S3
	N=136	N=39	N=55
PI-based	57 / 41.9	16 / 41.0	24 / 43.6
IMiD-based	56 / 41.2	17 / 43.6	24 / 43.6
PI- and IMiD-based	10 / 7.3	2 / 5.1	3 / 5.4
Cytotoxic agent-based	5 / 3.7	4 / 10.3	3 / 5.4
Other	6 / 4.4	0 / 0.0	1 / 1.8
No treatment required	2 / 1.5	0 / 0.0	0 / 0.0

Supplemental Table 4: Therapies at first disease relapse/progression

Available relapse therapies from n=230 are depicted. The overall p value for any difference between the applied relapse treatments between the three age groups (S1 vs. S2 vs. S3) is p=0.88.

Abbreviations: PI, proteasome inhibitor; IMiD, immunomodulatory agent.

Factor	TTP		NRM	
	HR (95% CI)	p	HR (95% CI)	p
Age group S2 (vs. S1)	1.30 (0.93-1.83)	0.13	1.83 (0.62-5.39)	0.28
Age group S3 (vs. S1)	0.96 (0.70-1.31)	0.79	2.14 (0.83-5.51)	0.11
Induction therapy (VCD)	0.96 (0.74-1.25)	0.77	-	-
Maintenance strategy (LEN-CR)	1.20 (0.93-1.55)	0.17	-	-
Sex (male)	1.35 (1.03-1.78)	**0.03**	-	-
ISS stage II	1.38 (1.01-1.88)	**0.04**	-	-
ISS stage III	1.47 (1.04-2.07)	**0.03**	-	-
LDH (>ULN)	1.55 (1.06-2.26)	**0.02**	-	-
Adverse cytogenetics (yes)	2.00 (1.51-2.65)	**<0.001**	-	-
IgA subtype (yes)	1.20 (0.89-1.62)	0.23	-	-
WHO PS (>1)	1.38 (0.90-2.12)	0.14	3.15 (1.15-8.60)	**0.03**
Progressive disease before HDM (yes)	1.32 (0.68-2.54)	0.41	-	-
Cardiac/vascular disorders (>1)	-	-	0.65 (0.19-2.27)	0.50

Supplemental Table 5: Multivariate model on time-to-progression and non-relapse mortality from transplantation

Age groups are defined as: ≤60 years (S1), 61-65 years (S2) and 66-70 years (S3). Adverse cytogenetics were defined as at least one of the following aberrations: deletion17p13, translocation t(4;14), translocation t(14;16), gain 1q21 (>3 copies).

Abbreviations: VCD, bortezomib, cyclophosphamide, dexamethasone; LEN, lenalidomide; CR, complete response; WHO, World Health Organization; PS, performance status; ISS, International Staging System; LDH, lactate dehydrogenase; ULN, upper limit of normal; Ig, immunoglobulin; HDM, high-dose melphalan.
