# Supplementary material for: Bortezomib-based induction, high-dose melphalan and lenalidomide maintenance in myeloma up to 70 years of age
Source: Leukemia. 2020 Jul 20;35(3):809–22. doi: 10.1038/s41375-020-0976-9 (PMC8318883; doi:10.1038/s41375-020-0976-9)
Supplement: Supplementary file 3 — Supplemental material 2 [file 41375_2020_976_MOESM3_ESM.pdf]

List of study sites in alphabetical order (main trial sites and associated trial sites), all sites are located in Germany

Facharztpraxis Hämatologie und Onkologie
Aschaffenburg

Praxisnetzwerk Hämatologie/ Internistische Onkologie
Bad Honnef

Facharztpraxis Onkologie
Bad Kreuznach

Caritas Krankenhaus Bad Mergentheim
Medizinische Klinik 2
Bad Mergentheim

MVZ Baden-Baden
Medizinisches Versorgungszentrum des Klinikums Mittelbaden
Baden-Baden

Charité Universitätsmedizin Berlin
Campus Benjamin Franklin
III. Medizinische Abteilung
Berlin

Charité Universitätsmedizin Berlin
Charité Campus Mitte
Medizinische Klinik mit Schwerpunkt Onkologie und Hämatologie
Berlin

HELIOS Klinikum Berlin-Buch
Klinik für Hämatologie, Onkologie und Immunologie
Berlin

Medizinisches Versorgungszentrum (MVZ)
Onkol. Schwerpunkt am Oskar-Helene-Heim
Berlin

Facharztpraxis Onkologie, Gastroenterologie, Hämatologie, Palliativmedizin
Berlin

Onkologie Seestrasse
Berlin

Klinikum Bielefeld Mitte
Klinik für Hämatologie, Onkologie und Palliativmedizin
Bielefeld

Facharztpraxis Onkologie, Gastroenterologie, Hämostaseologie, Palliativmedizin
Bochum

Universitätsklinikum Bonn
Med. Klinik und Poliklinik III
Schwerpunkte Onkologie, Hämatologie und Rheumatologie
Bonn

Zaho – Bonn
Zentrum für ambulante Hämatologie und Onkologie
Bonn

Johanniter Krankenhaus Bonn
Internistische Onkologie
Bonn

Onkologie Rheinsieg
Praxisnetzwerk Hämatologie/Internistische Onkologie
Bonn-Beuel

Städt. Klinikum Braunschweig
Medizinische Klinik III
Braunschweig

Onkologische Schwerpunktpraxis
Braunschweig

Onkologische Praxis im Krankenhaus Buchholz
Buchholz

Onkologische Schwerpunktpraxis
Celle

Klinikum Chemnitz
Innere Medizin III
Chemnitz

Regiomed Kliniken GmbH
Medizinisches Versorgungszentrum (MVZ) Coburg des Klinikums Coburg
Coburg

Carl-Thiem-Klinikum Cottbus
II. Medizinische Klinik
Cottbus

Klinikum Darmstadt
Medizinische Klinik V Hämatologie/Onkologie
Darmstadt

Onkologische Schwerpunktpraxis
Darmstadt

Gemeinschaftspraxis für Hämatologie und Onkologie
Medizinisches Zentrum am St.-Josefs-Hospital
Dortmund

Fachpraxis für Hämatologie und Onkologie

Erfurt

Universitätsklinikum Essen
Klinik für Hämatologie
Essen

Evangelisches Krankenhaus Essen-Werden Zentrum für Innere Medizin
Klinik für Hämatologie, Onkologie und Stammzell-transplantation
Essen

St. Antonius-Hospital
Klinik für Hämatologie und Onkologie
Eschweiler

Universitätsklinikum Frankfurt
Goethe-Universität
Medizinische Klinik II
Hämatologie, Onkologie, Rheumatologie, Infektiologie
Frankfurt am Main

Agaplesion Medizinisches Versorgungszentrum (MVZ) Frankfurt
Frankfurt am Main

Krankenhaus Nordwest
Klinik für Onkologie und Hämatologie
Frankfurt am Main

Interdisziplinäres Facharztzentrum (IFS) Frankfurt
Ambulantes Krebszentrum (AKS)
Frankfurt am Main

Vitanus GmbH
Frankfurt am Main

Frankfurter Rotkreuz-Kliniken
Klinik Maingau
Abt. Hämatologie/Onkologie und Palliativmedizin
Frankfurt am Main

PIOH-Praxis für Internistische Onkologie und Hämatologie
Frechen

Onkologische Facharztpraxis
Gerlingen

Facharztpraxis für Hämatologie und Onkologie
Gießen

Kath. Krankenhaus Hagen
St.-Josefs-Hospital
Klinik für Hämatologie und Onkologie
Hagen

Asklepios Klinik Altona
Abteilung Onkologie mit Sektion Hämatologie
Hamburg

Asklepios Klinik St. Georg
Abteilung Hämatologie, Onkologie und Stammzelltransplantation
Hamburg

Hämatologisch-Onkologische Praxis Altona (HOPA)
Hamburg

Facharztpraxis für Hämatologie und Onkologie
Hamburg

OncoResearch Lerchenfeld UG
Hamburg

Evangelisches Krankenhaus Hamm gGmbH
Medizinische Klinik
Hämatologie / Onkologie
Hamm

Onkologische Schwerpunktpraxis
63450 **Hanau**

Klinikum Hanau GmbH
Medizinische Klinik III
63450 **Hanau**

Klinikum Region Hannover
Klinikum Siloah
Onkologie und Palliativmedizin
Hannover

Onkologisches Ambulanzzentrum OAZ Hannover
Hannover

Universitätsklinikum Heidelberg
Medizinische Klinik V
Heidelberg

Onkologische Schwerpunktpraxis
Heidelberg

Onkologische Schwerpunktpraxis Heilbronn
Heilbronn

SLK Kliniken Heilbronn GmbH
Medizinische Klinik III
Heilbronn

Facharztpraxis für Hämatologie und Tumorerkrankungen
Henningsdorf

Universitätsklinikum des Saarlandes
Innere Medizin I
Homburg/Saar

Klinikum Idar-Oberstein
Medizinische Klinik I
Idar-Oberstein

Westpfalz-Klinikum
INN1
Kaiserslautern

Schwerpunktpraxis für Hämatologie und Onkologie
Kaiserslautern

Gemeinschaftspraxis für Hämatologie, Onkologie und Infektiologie
Karlsruhe

Institut für Versorgungsforschung in der Onkologie (InVo)
Koblenz

Universitätsklinikum Köln
Klinik I Innere Medizin
Köln

Onkologie Köln
Gemeinschaftspraxis für Onkologie und Hämatologie
Köln

Praxis Internistischer Onkologie und Hämatologie (PIOH)
Köln

Kliniken Köln, Krankenhaus Köln-Holweide
Köln

Facharztpraxis für Hämatologie, Onkologie und Gerinnung
Kronach

Onkologisches Zentrum Lebach
Caritaskrankenhaus Lebach
Lebach

Klinikum der Stadt Ludwigshafen am Rhein
Medizinische Klinik A
Ludwigshafen a. Rh.

Onkologische Schwerpunktpraxis Lüneburg
Lüneburg

Universitätsmedizin der Johannes Gutenberg-Universität Mainz
III. Medizinische Klinik
Mainz

MED Facharztzentrum
Gemeinschaftspraxis für Hämatologie und Onkologie
Mainz

Universitätsmedizin Mannheim
III. Medizinische Klinik
Hämatologie und Internistische Onkologie
Mannheim

Mannheimer Onkologie Praxis
Mannheim

Facharztpraxis für Innere Medizin, Hämatologie und Onkologie
Mannheim

Praxis für Innere Medizin, Hämatologie und internistische Onkologie
Marburg

Mühlenkreiskliniken (AöR)
Johannes Wesling Klinikum Minden
Hämatologie/Onkologie, Hämostaseologie und Palliativmedizin
Minden

Kliniken Maria Hilf
Krankenhaus St. Franziskus
Klinik für Hämatologie, Onkologie und Gastroenterologie
Mönchengladbach

Städtisches Klinikum München
Klinikum Harlaching
Klinik für Hämatologie, Onkologie und Palliativmedizin
München

Facharztpraxis für Innere Medizin, Hämatologie und Onkologie
Neunkirchen

medius Kliniken
Klinik Nürtingen
Klinik für Innere Medizin
Onkologie, Hämatologie
Nürtingen

Onkologische Facharztpraxis
Oberhausen

Paracelsus Kliniken
Klinik Osnabrück
Innere Medizin / Hämatologie und Onkologie
Osnabrück

medius Kliniken
Klinik Ostfildern-Ruit

Innere Medizin, Gastroenterologie
und Tumormedizin
Ostfildern

Medizinisches Versorgungszentrum
am Siloah St. Trudbert-Klinikum
Pforzheim

Onkologische Praxis Pinneberg
Pinneberg

Gemeinschaftspraxis Innere
Medizin/Onkologie
Pirmasens

Facharztpraxis für Onkologie
Rosenheim

Diakonie-Klinikum Schwäbisch Hall
Klinik für Innere Medizin III
Schwäbisch Hall

Zaho-Zentrum für ambulante
Hämatologie und Onkologie
Siegburg

Diakonie Klinikum Jung-Stilling
Innere Medizin
Siegen

Gastroenterologie Onkologie
Bodensee
Praxis Singen
Singen

Onkologische Schwerpunktpraxis
Speyer
Speyer

Marienhospital Stuttgart
Zentrum für Innere Medizin III
Onkologie, Hämatologie,
Palliativmedizin-
Stuttgart

Krankenhaus der Barmherzigen
Brüder Trier
Innere Medizin I
Trier

Klinikum Mutterhaus der
Borromäerinnen Trier
Innere Medizin I
Trier

Onkologische Schwerpunktpraxis am
Brüderkrankenhaus
Trier

Praxis für Innere Medizin,
Nephrologie, Hämatologie und
Onkologie
Trier

Onkologie Rheinsieg
Praxisnetzwerk Hämatologie und
Internistische Onkologie
Troisdorf

Universität Tübingen
Medizinische Klinik
Abtl. II/Hämatologie, Onkologie,
Immunologie und Rheumatologie
Tübingen

OMM Optimed Mundial GmbH
Viersen

Facharztpraxis für Onkologie
Wendlingen/Esslingen

Ammerland Klinik
Medizinische Klinik
Westerstede

Facharztpraxis für Onkologie
Westerstede

Onkologische Schwerpunktpraxis
Wolfsburg – Helmstedt
Praxis Wolfsburg
Wolfsburg
